# Understanding the Effect of Waiting for the Dissolution of Sodium Hydroxide in Geopolymer Concrete Mixes

**DOI:** 10.3390/ma18040849

**Published:** 2025-02-15

**Authors:** Samara Altameemi, Blessing O. Adeleke, John M. Kinuthia, Jonathan Oti

**Affiliations:** Faculty of Computing, Engineering and Science, University of South Wales, Pontypridd CF37 1DL, UK

**Keywords:** geopolymer concrete mix procedure, alkaline activator, ground granulated blast furnace slag (GGBS)

## Abstract

Geopolymer concrete (GPC) can be produced by the chemical activation of industrial by-products and processed natural minerals that contain aluminosilicates with the presence of an alkaline activator. Raw components are one of the critical parameters affecting geopolymer performance. On the other hand, the mixing procedure of geopolymer concrete is not any less important. Few demonstrative constructions have been built using GPC as a greener alternative to Portland cement concrete. Numerous variables affect GPC manufacture, such as raw material specification, activator type and dosage, and curing regimes. Despite the conventions of the building industry, the lack of proper mix design methods limits the wide acceptance of GPC in the industry. This report conducted experimental trials on GGBS-based GPC to optimize a mixing design procedure to achieve best mechanical strength and structural integrity. Geopolymer concrete properties were evaluated through slump and unconfined compressive strength tests. The laboratory trials in this report revealed that all geopolymer mixes, except SD0HV and 1W-SG, exhibited high workability values. Also, the presence of an alkaline activator was vital to attain satisfactory compressive strength values. The alkaline activator could be used when cooled and reached room temperature after two hours of preparation and was not necessary after 24 h. Mix G-(0.5W-S) with a 0.5A.A. (alkaline activator)/precursor (GGBS) ratio, SSA (sodium silicate alternative)/SH (sodium hydroxide with 10 M molarity) ratio of 1:1, and 0.55 W/B (water to binder) ratio is recommended to achieve best mechanical performance and structural integrity.

## 1. Introduction

The Portland cement industry is an important sector that helps the construction industry make concrete and other cement-based products. Apart from water, concrete is the most widely consumed product in the world (Scrivener et al., 2018) [1]. The widespread and continuous use of ordinary Portland cement (OPC) contributes towards pollution due to the association of carbon dioxide emissions (Rodríguez-Benavides et al., 2024) [2]. Cement manufacturing is responsible for approximately 7% of total global CO_2_ emissions (Supriya et al., 2023) [3]. Globally, there are escalating concerns regarding mismanagement in waste disposal, limited landfill spaces, and depletion of natural resources, which have become critical environmental issues that significantly impact global warming and climate change. Due to the seriousness of such matters, the demand has increased for exploring more eco-friendly and greener alternatives to ordinary Portland cement by using agricultural and industrial by-products for concrete production (Huseien et al., 2023) [4]. Some of the most common industrial by-products used in the production of geopolymer concrete are fly ash (FA), ground granulated blast furnace slag (GGBS), metakaolin (MK), and palm oil fuel ash (POFA). The utilization of these materials will not only tackle the issue of waste management and reducing landfills, it will also contribute towards energy saving, planet protection, and conservation of natural resources (Huseien et al., 2021) [5]. Geopolymers have been introduced as a promising greener alternative to OPC, with an astounding 90% lower CO_2_ footprint. Other benefits include less energy consumption and the use of waste as raw materials (Garces et al., 2021) [6].

Geopolymer concrete can be formed by activating aluminosilicate by-product waste material as a precursor with an alkaline activator solution. Unlike cement-based cementitious materials, various geopolymer systems possess different geopolymerization mechanisms, microstructure mechanical properties, and durability due to the diversity of the raw materials used (precursors) (Huang et al., 2022) [7]. Based on their calcium content, geopolymer precursors can be divided into two types: low-calcium systems and high-calcium systems. For low-calcium systems (e.g., fly ash), geopolymers require a highly alkaline environment for activation, and their main hydration products are zeolite-like and sodium aluminum silicate hydrate (N-A-S-H) gels, which form a three-dimensional framework by linking silicate tetrahedra and aluminum octahedra through shared oxygen atoms. For high-calcium systems (e.g., GGBS), geopolymers can be activated in a moderately alkaline environment, and their main products are calcium aluminum silicate hydrate (C-A-S-H) gels with chain-like forms and a low Ca/Si ratio (Zhang et al., 2024) [8]. In general, both types of gels can cohabit in geopolymer composite systems. With the increase in the calcium content in the precursor, the reaction products may transform from N-A-S-H gels into C-A-S-H gels. The ultimate chemical reaction in the formation of geopolymer concrete results in a three-dimensional network of aluminosilicate bonds, referred to as geopolymerization (Zhang et al., 2024) [8]. Meanwhile, sodium hydroxide (NaOH) and sodium silicate are used to induce the alkaline solution; the alkaline activator dissolves the aluminosilicate material and breaks down the aluminosilicate into smaller reactive species, primarily silicate (SiO_2_) and aluminum (AlO_4_) ions. The production of calcium aluminosilicate hydrate (C-A-S-H), sodium aluminosilicate hydrate (N-A-S-H), and calcium silicate hydrate (C-S-H) gels depends on the chemical composition or curing regimes (Bodur et al., 2023) [9].

The utilization of geopolymer is considered one of the best contributions to achieving green and sustainable construction engineering due to its superior mechanical properties and durability performance (Lingyu et al., 2021) [10], and it has been proven that geopolymer performs better than the Portland cement concrete (PCC) under the same testing conditions, such as chemical resistance (Adediran et al., 2022) [11], high-temperature strength (Luhar et al., 2021) [12], resistance to chloride penetration (Shi et al., 2021) [13], and the freeze–thaw cycle (Fu et al., 2011) [14]. On the other hand, there are some issues in practical application, such as (1) effective evaluation of waste source materials; (2) poor workability due to a sticky and thick mortar because of the high viscosity of the alkaline activator; (3) cost-intensive when using alkali silicate as an activator; (4) efflorescence in the final products due to the movement of unreacted alkalis in contact with water (Zhang et al., 2018) [15].

Many GPC studies show that most of the research is concentrated on the alkaline activator to binder ratio (Al/B), the water to solid ratio (w/s), the type and dosage of alkalis, the molar ratio in sodium silicate solutions, and curing conditions (Asghar et al., 2023) [16], but knowledge regarding the mixing time and mixing order of geopolymer precursors and components are very scarce (Ranjbar et al., 2020) [17]. Mahmood et al. (2021) [18] conducted field trials investigating the effect of the mixing duration on geopolymer concrete performance. The results showed that a long mixing time provides geopolymer concrete with a denser structure and superior mechanical properties, which can be attributed to the continuous dissolution of silicate and aluminate molecules from the precursor in an alkaline solution, which induces polycondensation and the formation of geopolymer chains (Gao et al., 2019) [19]. The inventor of geopolymer, Davidovits, reported that the manufacturing process of geopolymer concrete must follow the geopolymerization reaction kinetics to obtain maximum efficiency (Davidovits et al., 2020) [20]. The order in which the materials are added to the mix is significant as the alkali silicate should be given enough time to depolymerize the aluminosilicate precursor; following a correct order is crucial to ensure that the alkali silicate is given sufficient time to react with each component. If a more reactive ingredient is blended first, it risks absorbing more alkali silicates than it needs, which could create a deficit of alkali silicate for the remaining components and provide a slower or incomplete reaction.

This study was performed to establish the best mixing regime for the ground granulated blast furnace slag (GGBS)-based geopolymer concrete for adoption in practice by reducing the mixing period to one day, preferably very close to current practices. Two approaches were suggested to achieve the project aim. The first approach was proposed to optimize the preparation of the alkaline activator; it involved varying the time scale required for the alkaline activator to cool and react. The second approach was suggested to optimize the mixing order of the geopolymer concrete (GPC) ingredients; it involved differentiating the order in which the geopolymer concrete ingredients were added in the mixing procedure. Obtaining an efficient mixing procedure of the GGBS-based geopolymer concrete will tackle the waste management issue, conserve natural resources, and reduce greenhouse gas emissions through enhancing and widening the practical acceptance of geopolymer concrete applications.

## 2. Materials

The materials used in geopolymer preparation play a vital role in the geopolymer concrete performance and properties, thus a comprehensive analysis of these materials is crucial. The materials used in this study include ground granulated blast furnace slag (GGBS) as a precursor, NaOH pellets, silica fume, sand, aggregate (10 mm and 20 mm), and water. The GGBS in this study was used as an aluminosilicate precursor material and was manufactured and supplied by Tarmac CRH, Newport, UK, in accordance with British standard BS EN 15167-1:2006 [21].

The silica fume (SF) used in this study was a fine grey powder, undensified with a purity of 97.1%, supplied by Elkem, Sheffield, UK. The alkaline activator (AA) used consisted of sodium silicate alternative (SSA) and sodium hydroxide solution (NaOH). Two portions of sodium hydroxide (NaOH) solutions with a molarity of 10 M were prepared. One portion was used to activate the silica (SiO_2_) provided by the silica fume (SF) to obtain the sodium silicate alternative (SSA), which plays a vital role in the geopolymerization process. Each portion of the sodium hydroxide (NaOH) solution was prepared by dissolving the sodium hydroxide (NaOH) pellets in de-ionized water, as described by Billong et al. (2018) [22]. The NaOH used in this study was in the form of white laboratory-grade pellets with a purity of 98%, supplied by Fisher Scientific Ltd., Loughborough, Leicestershire, UK. The coarse aggregate used was limestone aggregate, specifically sized at 10 mm and 20 mm, while the fine aggregate was natural river sand extracted from the Bristol Channel, UK. The fine and coarse aggregates used were supplied by Jewson for building materials, Caerphilly, Wales, UK, in accordance with BS EN 12620:2002+A1:2008 [23].

Table 1 shows the oxide composition of GGBS and silica fume (SF). In addition, Table 2 contains the physical characteristics of GGBS and SF. Table 3 also presents some physical properties of the coarse and fine aggregates.

## 3. Methodology

### 3.1. Mix Design

This study was conducted to establish the optimal mixing regime of the ground granulated blast furnace slag (GGBS)-based geopolymer concrete for adoption in practice by reducing the mixing period to one day, preferably very close to current practices. Two approaches were suggested to achieve this study’s aim. The first approach focused on optimizing the preparation of the alkaline activator, and the second focused on optimizing the mixing order of the geopolymer concrete (GPC) ingredients.

Although all the mixes in both approaches followed the same mix design, the mixing procedure differed for each mix. The mix design chosen for this work was based on previous optimized lab work (Billong et al., 2021) [25]. The mix design comprised GGBS as the primary precursor used in the mix. A binder/sand/aggregate ratio of 1:2:3 and a 0.55 water-to-binder ratio were followed for all mixes. The alkaline activator comprised a sodium silicate alternative (SSA) and NaOH solution with a 1:1 volume ratio. The activator/binder ratio was 0.5.

#### 3.1.1. Approach 1: Optimizing the Preparation of the Alkaline Activator

This approach was performed to optimize the preparation of the alkaline activator by varying the time scale required for the preparation. The alkaline activator plays a crucial role in the preparation and properties of geopolymer concrete. Different objectives were suggested for achieving Approach 1, as shown in Table 4.

Table 5 shows the material proportions of the various mixes of Approach 1.

#### 3.1.2. Approach 2: Optimizing the Mixing Order of the Geopolymer Concrete (GPC) Ingredients

The second approach focused on optimizing the addition order of the GPC ingredients. The results from Approach 1 showed that the alkaline activator reacted well and showed satisfactory compressive strength values at 7, 28, and 90 days after cooling for 2 h; therefore, it was suggested to prepare the NaOH solution and the other slurries in the Approach 2 mixes and keep them for 2 h to react, hoping to achieve the same if not better mechanical properties.

Different objectives were suggested for achieving Approach 2, as shown in Table 6.

Table 7 shows the material proportions of the various mixes of Approach 2.

### 3.2. Alkaline Activator Preparation

The alkaline activator was obtained by mixing the sodium hydroxide (NaOH) solution with the sodium silicate alternative (SSA) at a 1:1 volume ratio. Based on the previous research, this ratio was considered a common ratio to be used to achieve satisfactory geopolymer concrete properties (Liu et al., 2019) [26]. Two portions of NaOH solutions with a molarity of 10 M were prepared for this study. Each portion was obtained by dissolving 400 g of NaOH pellets in 1 L of water.

The sodium silicate alternative (SSA) was obtained by mixing one portion of NaOH solution with undensified silica fume (SF) with a purity of 97.1%, as demonstrated in Equation (2), where two moles of sodium hydroxide (NaOH) in a liquid form react with two moles of silicon dioxide (SiO_2_) in a solid form to form one mole of sodium disilicate (Na_2_O(SiO_2_)_2_) and one mole of water (H_2_O) in a liquid form.

2SiO_2_ + 2NaOH → Na_2_O(SiO_2_)_2_ + H_2_O (1)

The sodium silicate alternative (SSA) and the other portion of the sodium hydroxide (NaOH) solution were kept in separate sealed containers for different periods to react, as previously explained in Approach 1, before adding to the dry geopolymer ingredients at a 1:1 volume ratio.

For Approach 2, not all mixes contained an alkaline activator, as the geopolymer ingredients were added in a different order to the mix, as previously explained in Approach 2. The alkaline activator is a critical component in the geopolymerization process; it plays a vital role in activating the aluminosilicate precursor. Sodium hydroxide was used to induce the silicate provided by the silica fume. This reaction is exothermic, and its heat is crucial for kickstarting geopolymerization.

### 3.3. Geopolymer Concrete Specimen Preparation and Testing Methods

The mixing procedure was performed to prepare the geopolymer concrete specimens. In this study, the mixing procedure was the main variable for all the mixes for Approaches 1 and 2.

Approach 1 consisted of five mixes. All mixes required alkaline activator preparation as the first stage of the mixing procedure, except mix (SD0HV), which was prepared by adding all activator ingredients in various orders during mixing. The alkaline activator of the other mixes was prepared on the same day of mixing, except for the control mix (PD), as its alkaline activator was prepared 24 h prior to the mixing day. The alkaline activator of mix (SD0H) was prepared and used immediately without cooling. In contrast, the alkaline activator of mix (SD3H) was used after cooling for up to 3 h (when reaching room temperature), and the alkaline activator of mix (SD3HF) was kept in the freezer for up to 3 h.

The temperature of the alkaline activator constituents (NaOH and SSA solutions) was measured using a digital thermometer during their cooling phase. This monitoring took place during the preparation of the PD, SD0H, SD3H, and SD3HF mixes. This was carried out to investigate the impact of the alkaline activator temperature variation on the geopolymer performance.

The mixing process started by mixing the dry ingredients, including aggregate, sand, and GGBS, in the mixing machine for 2–3 min. Then, the alkaline activator and, finally, the remaining water content, were added. All the ingredients were continuously mixed for another 2–3 min until the mix became homogeneous and ready to be cast.

Based on the results obtained from Approach 1, which showed that the alkaline activator reacted well and showed satisfactory workability properties and compressive strength results at various curing ages (7, 28, and 90 days) after cooling for 2 h, Approach 2 mix designs were suggested. In Approach 2 mixes, it was suggested that the sodium hydroxide (SH) solution and the other slurries be prepared and kept reacting and cooling for 2 h.

Approach 2 consisted of six mixes; the mixes were cast to examine the effect of changing the addition order of the geopolymer ingredients. The control mix (SD) was the same as in Approach 1. The mixing procedure of (0.5W-SG) started by adding the aggregates first, followed by the SH solution (half of the water content + NaOH pellets). Finally, the GGBS and SF were dissolved in the other half of the water content. The mixing procedure of (1W-SG) started by first adding the aggregates, followed by the NaOH pellets, GGBS, and SF dissolved in the total water content. The mixing procedure of (G-(0.5W-S)) started by adding the aggregates and the GGBS, followed by the NaOH pellets and SF dissolved in half of the water content, and, finally, the other half of the water was added to the mix. Mix (G-1W-S) was prepared by adding the aggregates and the GGBS to the mix, followed by the SH solution (total water content + NaOH pellets), and the SF was added at the end. The last mixing procedure (G-(1W-S)) was started by adding the aggregates and GGBS, followed by NaOH pellets and SF dissolved in the total water content.

The fresh properties of the geopolymer concrete were assessed using the slump test in accordance with British standard BS EN 12350-2:2019 [27]. Next, nine cubic specimens (100 mm × 100 mm × 100 mm) were cast for each mix composition in accordance with British standard BS EN 206:2013 [28]. After 24 h, the specimens were demolded and cured in a container maintained under controlled moist conditions, where temperature and humidity were kept constant. The hardened geopolymer concrete properties were examined by the unconfined compressive strength test at 7, 28, and 90 days in accordance with British standard BS EN 12390-3:2019 [29]. The cubic specimens were crushed (UCS test) at a rate of 6 kN/s. The reported results for the UCS are the average values obtained from two cubic specimens for each mix composition tested at the specified curing ages of 7, 28, and 90 days.

## 4. Results and Discussion

The specimens of Approach 1 and 2 mixes were tested for workability using the slump cone test in accordance with British standard BS EN 12350-2:2019 [27] and for unconfined compressive strength at 7, 28, and 90 days according to British Standard BS EN 12390-3:2019 [29]. In Approach 1, the temperature of the alkaline activator constituents (SH and SSA solutions) was monitored during cooling.

### 4.1. Alkaline Activator Temperature (Approach 1)

The alkaline activator was prepared for mixes PD, SD0H, SD3H, and SD3HF in Approach 1. SD0HV did not require alkaline activator preparation beforehand. The temperature of the NaOH and SSA solutions was monitored regularly during cooling to investigate the effect of the alkaline activator temperature variation on the geopolymer concrete performance. Table 8 shows the temperature of the NaOH and SSA solutions at various times.

It was observed from Table 7 that the temperature of the NaOH solution in all Approach 1 mixes increased significantly after adding the silica fume; this can be attributed to the start of the chemical reaction, which results in the formation of the sodium silicate alternative (SSA) and water as shown in Formula (2):

2SiO_2_ + 2NaOH → Na_2_O(SiO_2_)_2_ + H_2_O (2)

The chemical reaction between NaOH and SiO_2_ is exothermic, meaning that heat is released due to breaking bonds in NaOH and SiO_2_ and forming new bonds to produce SSA and water. The energy released during the bond formation is greater than the energy required to break the original bonds, leading to the net release of energy and a rise in solution temperature (Alnahhal et al., 2024) [30].

Moreover, a noticeable variation in the NaOH and SSA solutions’ temperatures in Approach 1 mixes was observed during mixing. In the PD mix, both the NaOH and SSA solutions were at room temperature during mixing, as they had been allowed to cool for 24 h. In contrast, in the SD0H mix, the temperatures of the NaOH and SSA solutions were 91 °C and 103.4 °C, respectively, since they were used immediately after preparation. Meanwhile, in the SD3H mix, the NaOH and SSA solutions were allowed to cool for 2 h before use, and their temperatures were observed to have dropped to 31 °C for the NaOH solution and 32 °C for the SSA solution, slightly above room temperature. The freezer was then used to further cool the NaOH and SSA solutions of the SD3HF mix, resulting in a significant reduction in temperature after being kept for up to 2 h in the freezer. The final observed temperatures were 15.5 °C for the NaOH solution and 13 °C for the SSA solution, both of which were below room temperature.

### 4.2. Workability

The fresh properties of Approach 1 and 2 specimens were tested using the slump cone test according to BS EN 12350-2:2019 [27].

**Figure 1:**
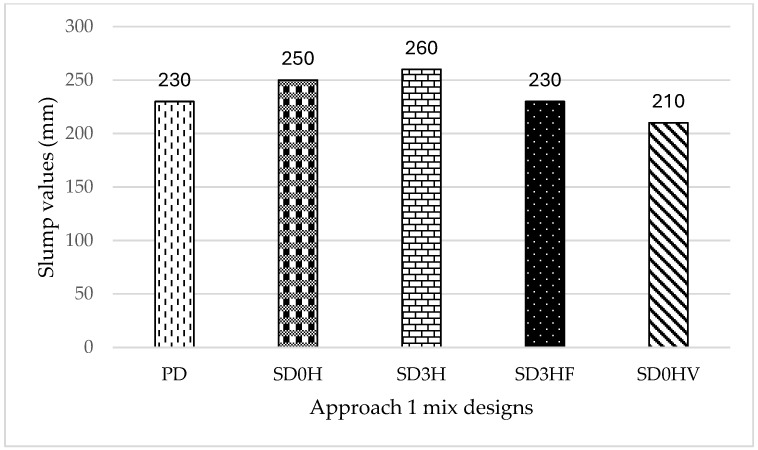
Slump data of Approach 1 mix designs (mm).

Figure 1 illustrates the slump values of the Approach 1 mix designs in (mm). Approach 1 mixes exhibited high workability properties ranging between 210 mm and 260 mm. Mix SD3HF exhibited a similar slump value as the control mix (PD), which was 230 mm. Both SD0H and SD3H revealed higher slump values than the control mix; however, the highest slump value was shown by mix (SD3H), which was equivalent to 260 mm, proving that the time needed for the alkaline activator to cool and react to achieve good workability properties is not necessarily 24 h, as it just needs sufficient enough time to cool and react to reach room temperature, which will vary depending on the surrounding atmosphere (in this case, it was 2 h). Mix (SD0HV) exhibited the lowest slump value due to the high segregation that occurred in the mix; this mix did not require alkaline activator preparation beforehand; instead, all the alkaline activator ingredients were added to the mix in various orders.

**Figure 2:**
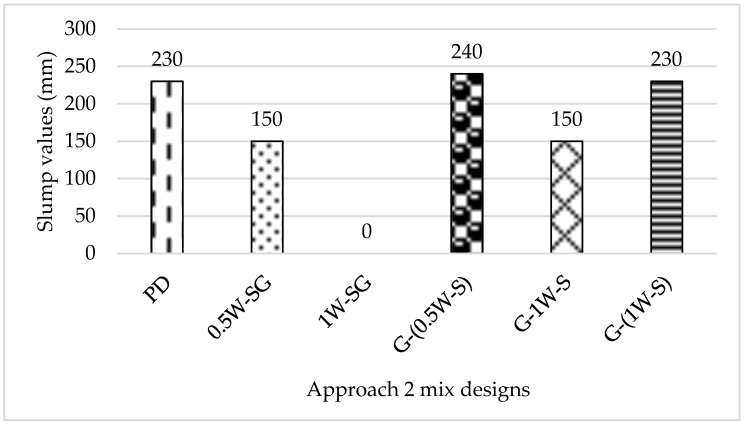
Slump data of Approach 2 mix designs (mm).

Figure 2 displays the slump data of the Approach 2 mix designs in (mm). Approach 2 mixes also showed satisfactory slump values ranging between 150 mm and 240 mm, except for mix (1W-SG), which failed at the first stage of preparation during chemical preparation. The solution of NaOH pellets plus SF and GGBS in the total water amount had solidified while being kept for 2 h to cool and react. This was attributed to the heat generated from the sodium hydroxide solution, which had kickstarted the hydration process with the presence of the GGBS (Korde et al., 2020) [31]. Both mixes (0.5W-SG) and (G-1W-S) exhibited good and similar slump values equivalent to 150 mm. Both mixes (G-(0.5W-S)) and (G-(1W-S)) showed slump values similar to the control mix; however, mix (G-(0.5W-S)) exhibited the highest slump value out of all Approach 2 mixes, with a value of 240mm and a sticky texture as if superplasticizers had been used, unlike mix (G-(1W-S)), which demonstrated a similar slump value (230 mm) but was not as sticky as mix (G-(0.5W-S)).

### 4.3. Unconfined Compressive Strength

The test used to determine the mechanical properties of Approaches 1 and 2 in this lab work was the unconfined compressive strength test. The cubic specimens of Approaches 1 and 2 were tested for compressive strength at 7, 28, and 90 days according to British Standard BS EN 12390-3:2019 [29].

**Figure 3:**
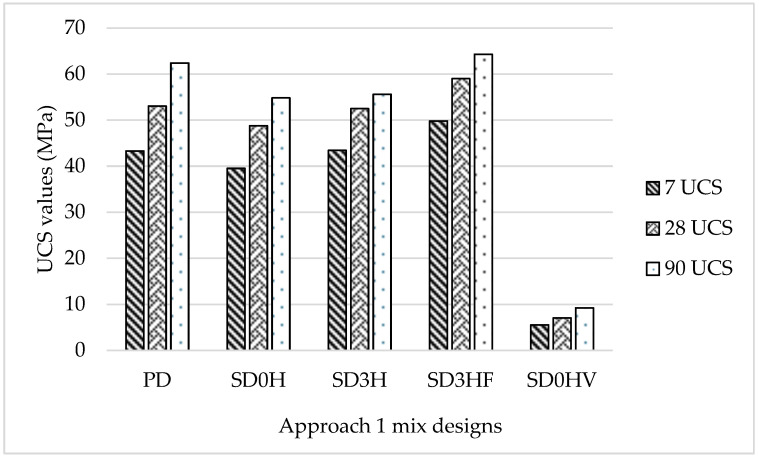
Compressive strength values of Approach 1 mix designs at 7, 28, and 90 days (MPa).

Figure 3 shows the unconfined compressive strength (UCS) values of Approach 1 mix designs at 7, 28, and 90 days in accordance with BS EN 12390-3:2019 [29]. The results showed that all Approach 1 mixes consisting of alkaline activators (PD, SD0H, SD3H, and SD3HF) exhibited higher compressive strength values than those with no alkaline activator (SD0HV). This proves that the presence of an alkaline activator is substantial in the compressive strength development of geopolymer concrete. Mix (SD0HV) showed the lowest compressive strength value, which can be attributed to the mix’s high segregation and the absence of an alkaline activator. Mix (SD3H) exhibited a high value of compressive strength similar to the control mix (SD). The similarity of the compressive strength values in the control mix and SD3H proves that the time needed for the alkaline activator to react is not necessarily 24 h, as it can fully react and manifest satisfactory compressive strength values when its temperature reaches room temperature, which is nearly 2 h from preparation time depending on the surrounding atmosphere. The results also showed that compressive strength continued to increase with time; however, up to 64% of the compressive strength was achieved at 7 days, which can be attributed to the relatively high content of MgO in the GGBS. MgO contributes to the formation of the hydrotalcite (Mg_6_Al_2_(OH)_16_CO_3_), which plays a vital role in the early strength development of slag-based geopolymer concrete (Wang et al., 2020) [32].

**Figure 4:**
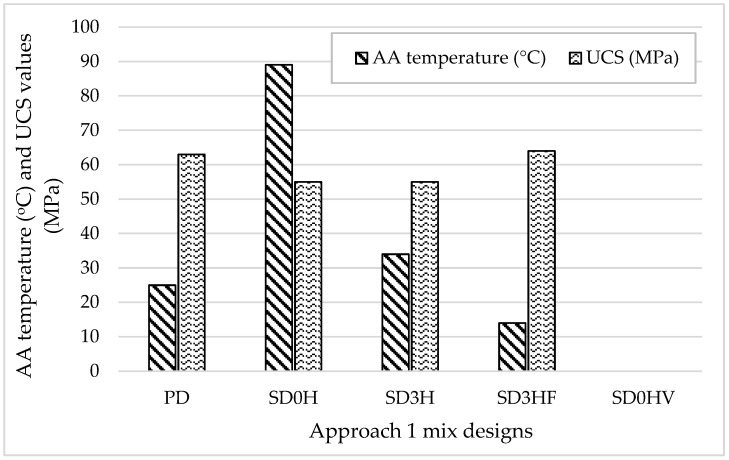
The relation between alkaline activator (AA) temperature (°C) and UCS (MPa) of Approach 1 mix designs.

Figure 4 demonstrates the relation between the alkaline activator temperature at mixing time and the unconfined compressive strength (UCS) of the Approach 1 mix designs. The compressive strength results of the Approach 1 mix designs clearly demonstrated an inverse relation between the alkaline activator temperature at mixing time and the compressive strength value at 90 days, as shown in Figure 4.

As the alkaline activator temperature decreased, the compressive strength value increased, which can be attributed to the acceleration of the geopolymerization reaction with higher temperatures, which can result in the formation of microstructural defects.

These defects, such as microcracks or an incomplete dissolution of aluminosilicate materials, can weaken the overall structure of geopolymer concrete (Özbayrak et al., 2023) [33].

**Figure 5:**
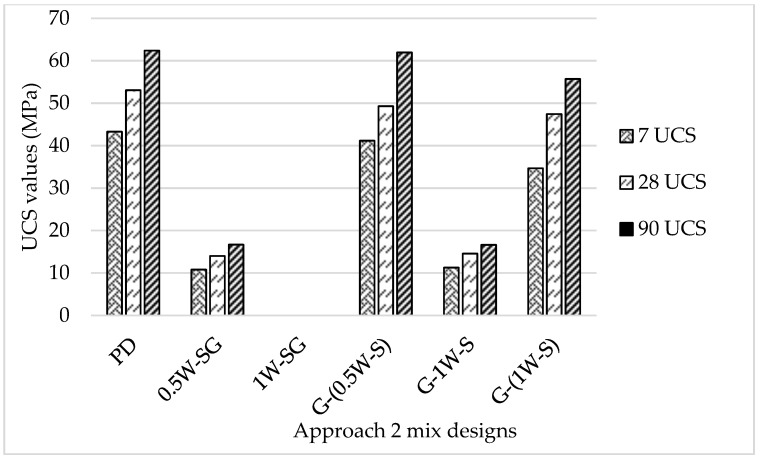
Compressive strength values of Approach 2 mix designs at 7, 28, and 90 days (MPa).

Figure 5 details the UCS performances of the concrete mixes in the Approach 2 observations. The compressive strength results obtained from the Approach 2 mixes varied significantly. Both mixes (G-(0.5W-S)) and (G-(1W-S)) exhibited relatively high compressive strength values, unlike mixes (0.5W-SG) and (G-1W-S), which showed considerably low compressive strength results. The variation in the compressive strength values in these mixes can be attributed to the presence of the alkaline activator in mixes (G-(0.5W-S)) and (G-(1W-S)), unlike the other two mixes (0.5W-SG) and (G-1W-S), which also consisted of the NaOH solution (but SF was added to the total mix rather than being dissolved in the SH solution). The alkaline activator is considered the key ingredient that initiates and sustains the geopolymerization process, leading to the formation of the strong gel matrix that is the backbone of geopolymer concrete. This gel matrix fills the voids between the aggregate particles, providing geopolymer concrete with high compressive strength and durability (Mohamed et al., 2024) [34]. Although both mixes (G-(0.5W-S)) and (G-(1W-S)) exhibited high compressive strength values, the compressive strength of mix (G-(0.5W-S)) was the closest to that of the control mix than mix (G-(1W-S)), which can be attributed to the higher concentration of NaOH and SF in (G-(0.5W-S)) than mix (G-(1W-S)). The higher concentration of sodium hydroxide and silica fume will enhance the dissolution and reactivity of the mix, as the high concentration of sodium hydroxide will increase the pH value of the solution, which will enhance the dissolution of the silica fume. This higher dissolution rate releases more reactive silica species into the solution, which leads to the formation of a greater amount of aluminosilicate gel during the geopolymerization process; this gel is crucial for the binding properties of the geopolymer matrix (Mohamed et al., 2024) [34].

The compressive strength of the Approach 2 mix designs continued to increase with time; however, up to 64% of the compressive strength was achieved at 7 days due to the relatively high content of MgO in the GGBS, which contributed to the formation of the hydrotalcite (Mg_6_Al_2_(OH)_16_CO_3_), which plays a significant role in the early strength development of slag-based geopolymer concrete (Wang et al., 2020) [32].

## 5. Conclusions

This current experimental study focused on establishing the best mixing regime for geopolymer concrete for adoption in practice by reducing the mixing period to one day, preferably very close to current practices, and optimizing the mixing order of the geopolymer concrete ingredients. Mix (G-(0.5W-S)) manifested the best mechanical strength and structural integrity. This study concentrated on establishing the best mixing regime for geopolymer concrete. While this is important, it limited the scope to one aspect of the geopolymer concrete process, potentially neglecting other factors that might influence the final properties of the material. The following conclusions are drawn:

▪ The use of an alkaline activator is crucial for the geopolymerization process; its temperature at the time of mixing needs to be controlled to ensure proper reaction kinetics, essential for achieving optimal compressive strength in geopolymer concrete.
▪ The temperature of the SH solution increased significantly after adding SF due to the start of the exothermic chemical rection to form the new bonds of the sodium silicate alternative and water.
▪ All mixes exhibited satisfactory workability properties ranging between 150 mm and 260 mm, except those that did not contain the NaOH solution (SD0HV and 1W-SG).
▪ High segregation was shown by mix (SD0HV), where no alkaline activator preparation was required beforehand; instead, the geopolymer ingredients were added to the mix in various orders.
▪ Mixing the total water content with the NaOH pellets, GGBS, and SF caused mix (1W-SG) to fail at the first stage (during chemical preparation) due to the early start of the hydration process, with the presence of GGBS aided by the heat generated from the SH dissolution in water.
▪ The highest slump value was achieved in mix (SD3H), which contained an alkaline activator with a temperature equivalent to room temperature after cooling for 2 h.
▪ The alkaline activator used to prepare GPC with acceptable fresh and hardened properties does not necessarily need 24 h to fully reach; it can be used when it has cooled and reached room temperature.
▪ An inverse relation was observed between the alkaline activator temperature at mixing time and the compressive strength value—as the alkaline activator temperature decreased, the compressive strength value increased.
▪ The mixes consisting of alkaline activators achieved the highest compressive strength values despite the varying concentrations of NaOH and SF in the mix.
▪ The compressive strength values of all mixes continued to increase with time.
▪ Up to 64% of the compressive strength was achieved at 7 days due to the relatively high content of MgO in the GGBS, which contributed to the formation of the hydrotalcite (Mg_6_Al_2_(OH)_16_CO_3_), which plays a significant role in the early strength development of slag-based geopolymer concrete (Wang et al., 2020) [32].
▪ Mix G-(0.5W-S) (which was formulated by adding the aggregates + GGBS, then half of the water mixed with NaOH pellets + SF, and then the other half of water) was considered the optimized mix in terms of its mechanical performance and properties.

## Figures and Tables

**Table 1:** Oxide composition of GGBS and SF (Adeleke et al., 2023) [24].

Oxide	Composition (wt %)	
	GGBS	SF
SiO_2_	35.54	97.1
Al_2_O_3_	11.46	0.2
Fe_2_O_3_	0.42	0.1
Na_2_O	0.37	-
CaO	37.99	0.2
MgO	8.78	0.1
K_2_O	0.43	0.2
TiO_2_	0.7	-
V_2_O_5_	0.04	-
P_2_O_5_	0.02	0.03
SO_3_	1.54	0.1
Mn_2_O_3_	0.43	-
BaO	0.09	-
L.O.I	2.00	0.5

**Table 2:** Physical properties of GGBS and SF (Adeleke et al., 2023) [24].

Property	GGBS	SF
Specific gravity	2.9	3.15
Bulk density (kg/m^3^)	1200	300
Insoluble residue	0.3	-
Glass content (%)	90	-
Blaine fineness (m^2^/kg)	450	-
Alkalinity value (pH)	10.4	7
Color	Off-white	Grey
Physical form	Fine powder	powder

**Table 3:** Some physical properties of the coarse and fine aggregates.

Physical Properties	Coarse Aggregate		Fine Aggregate (Sand)
	20 mm	10 mm	
Uniformity coefficient (CU)	1.3	3.3	0.11
Curvature coefficient (CC)	7.5	1.5	1.75
Flakiness index (%)	23	30–35	-
Elongation index (%)	12	17–22	-
Shape index (%)	7	12	-
Impact value	15	23	-
Fineness modulus (mm)	-	4	1.54
Uncompacted bulk density (kg/m^3^)	2570	1350	1500
Pre-dried particle density (kg/m^3^)	-	2690	2600
Water absorption (%)	1.1	2	21

**Table 4:** Approach 1 objectives.

Mix	Description
PD	The alkaline activator was prepared 24 h prior to the mixing day.
SD0H	Reducing the activator preparation period from the previous day (24 h) to same day mixing, immediately before mixing without cooling.
SD3H	Reducing the activator preparation period from the previous day (24 h) to same day mixing, up to 3 h prior to mixing.
SD3HF	Reducing the activator preparation period from the previous day (24 h) to same day mixing, aided by cooling in the freezer for up to 3 h.
SD0HV	Reducing the activator preparation period from the previous day (24 h) to adding all activator ingredients in various orders during mixing.

**Table 5:** Approach 1 mix proportions.

Mix Design	Alkaline Activator					Precursor (kg)	Sand (kg)	Aggregate (kg)		Water (W3) (L)
	SSA			SH Sol. (kg)						
	SH Sol. (mL)		SF (kg)							
	Water (W1) (L)	SH Pellets (kg)		Water (W2) (L)	SH Pellets (kg)			10 mm	20 mm	
PD, SD0H, SD3H, SD3HF	0.455	0.182	0.281	0.455	0.182	2.67	8	4	8	1.29
SD0HV	0	0	0.281	0	0.364	2.67	8	4	8	2.2

**Table 6:** Approach 2 objectives.

Mix	Description
PD	The control mix, the same control mix prepared in Approach 1, consisted of an alkaline activator prepared 24 h prior to the mixing day.
0.5W-SG	Investigating the effect of changing the addition order of the GPC ingredients, starting by adding the aggregates, then the NaOH solution (half water amount + NaOH pellets), and then the mixture of SF and GGBS with the other half of water.
1W-SG	Investigating the effect of changing the addition order of the GPC ingredients, starting by adding the aggregates and then a solution of the total amount of water + NaOH pellets + GGBS + SF.
G-(0.5W-S)	Investigating the effect of changing the addition order of the GPC ingredients, starting by adding the aggregates + GGBS, then half of the water mixed with NaOH pellets + SF, and then the other half of water.
G-1W-S	Investigating the effect of changing the addition order of the GPC ingredients, starting by adding the aggregates + GGBS to the NaOH solution (total amount of water + NaOH pellets) and then adding SF at the end.
G-(1W-S)	Investigating the effect of changing the addition order of the GPC ingredients, starting by adding the aggregates + GGBS, then the slurry (total amount of water + NaOH pellets + SF).

**Table 7:** Approach 2 mix proportions.

Mix Design	Alkaline Activator					Precursor (kg)	Sand (kg)	Aggregate (kg)		Water (W3) (L)
	SSA			SH Sol. (kg)						
	SH Sol. (mL)		SF (kg)							
	Water (W1) (L)	SH Pellets (kg)		Water (W2) (L)	SH Pellets (kg)			10 mm	20 mm	
PD	0.455	0.182	0.281	0.455	0.182	2.67	8	4	8	1.29
0.5W-SG, G-(0.5W-S)	1.1	0.364	0.281	0	0	2.67	8	4	8	1.1
1W-SG, G-1W-S, G-(1W-S)	0	0.364	0.281	0	0	2.67	8	4	8	2.2

**Table 8:** Temperature of NaOH and SSA solutions at various times in °C (Approach 1).

Time	Temp. °C (PD)		Temp. °C (SD0H)		Temp. °C (SD3H)		Temp. °C (SD3HF)		Temp. °C (SD0HV)	
	NaOH	SSA	NaOH	SSA	NaOH	SSA	NaOH	SSA	NaOH	SSA
Prep. time (0 min.)	92	103	91	103.4	93	104.7	93	105	No alkaline activator	
30 min. after prep.	N/A	N/A	N/A	N/A	62	77	54	61	No alkaline activator	
60 min. after prep.	N/A	N/A	N/A	N/A	44.6	47.6	21.4	25.7	No alkaline activator	
90 min. after prep.	N/A	N/A	N/A	N/A	38	39.9	14.2	12.5	No alkaline activator	
Mixing time	room temp. (25 ± 2) (24 h)	room temp. (25 ± 2) (24 h)	73.2 (15 min)	88.6 (15 min)	31 (2 h)	32 (2 h)	15.5 (1 h and 45 min)	13 (1 h and 45 min)	No alkaline activator	

Note: N/A—not applicable.

## Data Availability

The original contributions presented in this study are included in the article. Further inquiries can be directed to the corresponding author.
